# Validation of rheumatoid arthritis diagnoses in health care utilization data

**DOI:** 10.1186/ar3260

**Published:** 2011-02-23

**Authors:** Seo Young Kim, Amber Servi, Jennifer M Polinski, Helen Mogun, Michael E Weinblatt, Jeffrey N Katz, Daniel H Solomon

**Affiliations:** 1 Division of Pharmacoepidemiology and Pharmacoeconomics, Brigham and Women's Hospital/Harvard Medical School, 75 Francis Street, Boston, MA 02115, USA; 2 Division of Rheumatology, Brigham and Women's Hospital, 75 Francis Street, Boston, MA 02115, USA; 3 Department of Orthopedic Surgery, Brigham and Women's Hospital, 75 Francis Street, Boston, MA 02115, USA; 4 Department of Epidemiology, Harvard School of Public Health, 677 Huntington Avenue, Boston, MA 02115, USA

## Abstract

**Introduction:**

Health care utilization databases have been increasingly used for studies of rheumatoid arthritis (RA). However, the accuracy of RA diagnoses in these data has been inconsistent.

**Methods:**

Using medical records and a standardized abstraction form, we examined the positive predictive value (PPV) of several algorithms to define RA diagnosis using claims data: A) at least two visits coded for RA (ICD-9, 714); B) at least three visits coded for RA; and C) at least two visits to a rheumatologist for RA. We also calculated the PPVs for the subgroups identified by these algorithms combined with pharmacy claims data for at least one disease-modifying anti-rheumatic drug (DMARD) prescription.

**Results:**

We invited 9,482 Medicare beneficiaries with pharmacy benefits in Pennsylvania to participate; 2% responded and consented for review of their medical records. There was no difference in characteristics between respondents and non-respondents. Using 'RA diagnosis per rheumatologists' as the gold standard, the PPVs were 55.7% for at least two claims coded for RA, 65.5% for at least three claims for RA, and 66.7% for at least two rheumatology claims for RA. The PPVs of these algorithms in patients with at least one DMARD prescription increased to 86.2%-88.9%. When fulfillment of 4 or more of the ACR RA criteria was used as the gold standard, the PPVs of the algorithms combined with at least one DMARD prescriptions were 55.6%-60.7%.

**Conclusions:**

To accurately identify RA patients in health care utilization databases, algorithms that include both diagnosis codes and DMARD prescriptions are recommended.

## Introduction

Large automated databases such as health care utilization and medical record databases have been widely used as data sources for epidemiologic studies [1]. Validity and completeness of prescription drug data in health care utilization databases with the prescription drug plan have been checked several times and reported as being of high quality [2], but the accuracy of specific disease data such as diagnosis of rheumatoid arthritis (RA) in health care utilization data has been somewhat questionable.

Several studies previously examined the accuracy of RA diagnoses in various data sources and reported inconsistent results [3-8]. A previous study examined the accuracy of computerized database diagnoses of RA among the Olmsted County residents in Minnesota on the basis of chart review and found a sensitivity of 89%, a specificity of 74%, and a positive predictive value (PPV) of 57% by using the American College of Rheumatology (ACR) RA criteria as the gold standard [3]. The PPV of the RA diagnosis codes alone was only 66% compared with the gold standard definition of RA diagnosis by a rheumatologist on two separate visits in a study using the Minneapolis Veterans Affairs administrative data [7]. A Danish national register-based study showed that 59% of the subjects identified by the algorithm using only discharge diagnosis codes had a clinical diagnosis of RA and that 46% of those met the ACR criteria for RA [8].

However, the sensitivity and PPV were over 90% for the chart documentation of RA diagnosis in a study of Medicare diagnosis claims for RA from several rheumatology practices [4]. The PPV of the RA diagnosis codes from Medicare inpatient claims among total hip replacement recipients was 86% for the chart documentation of RA diagnosis [5]. Another administrative data-based algorithm with at least two physician visit claims for RA (with at least 30 days between the visits) had a PPV of 92% for RA based on a patient self-report questionnaire [6].

In this study, we developed several diagnosis code-based algorithms with and without a link to pharmacy claims for disease-modifying antirheumatic drugs (DMARDs) to define the outpatient diagnosis of RA in a health care utilization database and compared the validity of these algorithms to various gold standard definitions.

## Materials and methods

### Data source

We studied participants in the Pennsylvania Assistance Contract for the Elderly (PACE) program, established in 1984 to assist Pennsylvania residents who are 65 years or older, who are of low to moderate income, and who may suffer financial hardship in paying for their medication. The PACE program provides pharmacy benefits for all drugs, including DMARDs and biologic therapy, for qualifying residents who are 65 or older. All PACE participants receive Medicare benefits. Data use agreements were in place with Medicare and the PACE program that supplied information for the study database. This work was approved by Brigham and Women's Hospital's Institutional Review Board.

### Study procedures

Three different algorithms were used to identify patients with RA by using the Medicare claim data from 1994 to 2004: (a) beneficiaries with at least two claims associated with RA (International Classification of Diseases, 9th Revision, Clinical Modification [ICD-9 CM] code 714), (b) beneficiaries with at least three claims associated with RA, and (c) beneficiaries with at least two RA claims that were from a rheumatologist and that were separated by at least 7 days. All inpatient, outpatient, and procedure claims such as laboratory or radiologic tests were included. We identified rheumatologists with a Medicare provider specialty code in the database and verified them with the ACR membership directory. A subgroup of patients who filled at least one prescription for DMARDs over a period of 1 year after the RA diagnosis was then identified by using the data from both pharmacy benefit program and claim data for infusions. To compare baseline characteristics of the study subjects, we selected a group of beneficiaries who never had any claims for RA.

After identifying subjects by each of the algorithms, we attempted to obtain consent to review their medical record. First, the PACE program mailed a letter to the groups of subjects identified by our algorithms to inform them that they would be contacted by our research group. A letter that provided details about the study was then sent to the subjects in each of the groups and asked whether they would consent to have the study researchers review their medical records from their physicians, including doctors who treated them for arthritis. Subjects who agreed to participate in the study signed a consent and authorization form for release of medical records. Additionally, subjects were asked to complete a physician information form to identify their primary physicians as well as specialists and their contact information. We then attempted to obtain copies of medical records.

Once we received the medical records, all personal identifiers were removed from the records for protection of patients' privacy. Medical records were reviewed independently by several rheumatologists at Brigham and Women's Hospital. To minimize inter-reviewer variation in data abstraction, a structured data abstraction form was developed and pilot-tested with the principal investigator (DHS). The form included items such as the seven ACR 1987 classification criteria for RA, disease onset, other rheumatologic diagnoses, medications, and laboratory data. On the basis of these data, the reviewers assessed whether a patient met the gold standard definitions of RA: (a) diagnosis of RA by a rheumatologist and (b) fulfillment of the ACR criteria for RA. Any indication in the medical record that the diagnosing rheumatologists thought that the patient had RA at that time was counted as having 'RA diagnosis per rheumatologists'. When the patients were not seen by rheumatologists, 'RA diagnosis per rheumatologists' was made by the reviewers on the basis of the data from their medical records. When the diagnosis of RA was neither documented nor clear in their medical records, the patients were considered non-RA. Areas of disagreement or uncertainty were resolved by consensus. The study period for data collection from medical records lasted from 2004 to 2008.

### Statistical analyses

We calculated PPV as the percentage of the patients who met the gold standard definitions among those identified by the algorithms. We also examined the PPVs of these algorithms combined with at least one prescription fill for a DMARD (Table 1). Ninety-five percent confidence intervals (CIs) of the PPVs were calculated by using the normal approximation of the binomial distribution. All analyses were conducted with SAS 9.1 Statistical Software (SAS Institute Inc., Cary, NC, USA).

**Table 1:** A list of disease-modifying antirheumatic drugs included in the study

Abatacept
Adalimumab
Anakinra
Azathioprine
Cyclosporin
D-penicillamine
Etanercept
Gold
Hydroxychloroquine
Infliximab
Leflunomide
Methotrexate
Minocycline
Rituximab
Sulfasalazine

## Results

### Characteristics of the study population

A total of 9,482 patients were identified with the algorithms. Only 2% of the patients consented to have medical records reviewed for our study. Subsequently, medical records were obtained in 83.1% of those who consented to the study. Demographic characteristics were similar between respondents and non-respondents. Among the non-respondents, the mean age was 80.7 years with a standard deviation (SD) of 6.8, and 85.9% were female. Table 2 describes the characteristics of study subjects identified by each algorithm. Overall, the mean age was 79.3 (SD 7.1) years, 82.9% were female, and 98.2% were Caucasians. The patients identified by the algorithm requiring at least two claims from a rheumatologist were slightly younger and had more comorbidities than the patients identified by the other algorithms.

**Table 2:** Baseline characteristics of study subjects

Algorithms	A. At least 2 claims for RA	B. At least 3 claims for RA	C. At least 2 claims from a rheumatologist^a^	No claims for RA
Number	131	110	84	39
Age in years, mean (SD)	79.1 (6.7)	78.8 (6.6)	78.7 (7.0)	80.1 (8.4)
Females, number (percentage)	115 (87.8)	96 (87.3)	73 (86.9)	26 (66.7)
Caucasians, number (percentage)	129 (98.5)	109 (99)	83 (98.8)	38 (97.4)
Comorbidity index, mean (SD)	2.6 (2.3)	2.6 (2.3)	2.7 (2.4)	1.8 (2.5)
Comorbidity index >0, number (percentage)	109 (83.2)	92 (83.6)	72 (85.7)	20 (51.3)
Rheumatology visits, mean (SD)	1.9 (3.6)	2.2 (3.8)	3.0 (4.1)	0 (0)
DMARD use, number (percentage)	58 (44.3)	56 (50.9)	45 (53.6)	1 (2.6)

^a^At least 7 days were required between the claims. DMARD, disease-modifying antirheumatic drug; RA, rheumatoid arthritis; SD, standard deviation.

### Positive predictive value for various algorithms

Table 3 presents the PPV of each algorithm. When 'RA diagnosis per rheumatologists' was used as the gold standard, the PPVs were 55.7% (95% CI 46.8% to 64.4%) for the algorithm of at least two claims for RA and 65.5% (95% CI 55.8% to 74.3%) for the algorithm of at least three claims for RA. When the algorithm was restricted to at least two claims that were from a rheumatologist and that were separated by at least 7 days, the PPV increased to 66.7% (95% CI 55.5% to 76.6%). The PPVs of these algorithms were generally lower, ranging from 33.6% to 40.0%, with fulfillment of four or more of the ACR RA criteria as the gold standard.

**Table 3:** Positive predictive values and 95% confidence intervals of the algorithms to define rheumatoid arthritis in health care utilization data

Gold standard definition	A. At least 2 claims for RA	B. At least 3 claims for RA	C. At least 2 claims from a rheumatologist^a^
DMARD prescription filling is not required			
Number	131	110	84
RA per rheumatologists, number	73	72	56
PPV (95% CI)	55.7 (46.8-64.4)	65.5 (55.8-74.3)	66.7 (55.5-76.6)
At least 4 ACR criteria, number	44	44	33
PPV (95% CI)	33.6 (25.6-42.4)	40.0 (30.8-49.8)	39.3 (28.8-50.6)
At least 3 ACR criteria, number	56	56	42
PPV (95% CI)	42.8 (34.2-51.7)	50.9 (41.2-60.6)	50.0 (38.9-61.1)
At least 1 DMARD prescription filling is required			
Number	58	56	45
RA per rheumatologists, number	50	49	40
PPV (95% CI)	86.2 (74.6-93.9)	87.5 (75.9-94.8)	88.9 (76.0-96.3)
At least 4 ACR criteria, number	34	34	25
PPV (95% CI)	58.6 (44.9-71.4)	60.7 (46.8-73.5)	55.6 (40.0-70.4)
At least 3 ACR criteria, number	42	42	33
PPV (95% CI)	72.4 (59.1-83.3)	75.0 (61.6-85.6)	73.3 (58.1-85.4)

Positive predictive values (PPVs) are presented as a percentage. ^a^At least 7 days were required between the claims. ACR, American College of Rheumatology; CI, confidence interval; DMARD, disease-modifying antirheumatic drug; RA, rheumatoid arthritis.

When at least one DMARD prescription was required, the PPV improved to 86.2% (95% CI 74.6% to 93.9%) for the algorithm of at least two claims for RA, with 'RA diagnosis per rheumatologists' as the gold standard. The PPV was highest (88.9%, 95% CI 76.0% to 96.3%) for the algorithm of at least two claims from a rheumatologist combined with at least one DMARD prescription. When fulfillment of four or more of the ACR RA criteria was used as the gold standard, the PPVs of the algorithms combined with at least one DMARD prescription ranged from 55.6% to 60.7% (Table 3).

Less than 20% of the patients were identified with ICD-9 714.9, which is for unspecified inflammatory polyarthropathy. In a sensitivity analysis, we excluded those patients and recalculated the PPVs of the algorithms. Overall, the PPV did not improve substantially. The PPVs were 60.7% (95% CI 51.8% to 69.5%) for the algorithm of at least two claims for RA and 70.1% (95% CI 61.0% to 79.2%) for the algorithm of at least three claims for RA using 'RA diagnosis per rheumatologists' as the gold standard. The algorithm of at least two claims from a rheumatologist had the PPV of 73.0% (95% CI 62.9% to 83.1%).

## Discussion

This study examined the PPV of various algorithms for identifying patients with RA in health care utilization data and found that the diagnosis code-based algorithms had modest PPVs, ranging from 55.7% for the least restrictive algorithm to 66.7% for the most restrictive, using the diagnosis of RA by a rheumatologist as the gold standard. However, we found that requiring a DMARD prescription improved the PPVs substantially. We also found that PPVs were lower when fulfillment of four or more of the ACR RA criteria was used as the gold standard.

Previous studies of Medicare claim data for the RA diagnosis showed the high PPVs over 85% compared with the chart documentation of RA diagnosis [4,5]. The better performance of the RA diagnosis codes in these studies can be explained by a difference in patient population as these studies were limited to either a hospital inpatient setting for joint replacement surgery or rheumatology specialty clinics.

Our study has important implications. Based on our results, a diagnosis code-based algorithm alone is not sufficient to accurately identify patients with RA in the health care utilization data. Further refinement of the algorithms with a link to pharmacy claim data for a DMARD prescription can improve the PPVs of RA diagnoses in these data. Studies assessing RA-specific complications or the burden of RA solely on the basis of the ICD-9 code should be interpreted with caution.

Several limitations of this study should be noted. First, generalizability can be an issue with the low response rate, although we did not find a significant difference in demographic characteristics between respondents and non-respondents. We attempted to recruit as many patients as possible and sent multiple recruitment letters over a period of 3 years, but the response rate was only 2%. One of the main reasons for this low response rate is that this study required patients in the community to provide an authorization to release their medical records to the study investigators, who were not directly or indirectly involved in their medical care. Other potential explanations for such a low response rate include older age, low socioeconomic status, admission to a nursing home, critical illness, and death. Second, our focus on the elderly can be seen as a limitation as it is possible that validity may vary by age group as our study included only those patients who were 65 or older. However, the prevalence of RA among adults who are 60 years or older in the US is approximately 2% [9]; therefore, the elderly populations contain the substantial proportion of RA patients in the population. Third, the percentage of the patients who met the ACR criteria in our review was low. It might have been underestimated as we did not have access to all the longitudinal medical records across multiple physicians. Incompleteness of information that is needed to assess the fulfillment of the individual ACR RA criteria in medical records has been previously reported [10,11]. The diagnostic performance of the ACR classification criteria for RA is also known to be problematic in a clinical setting [12].

Our study demonstrated that the PPVs of RA diagnosis codes in the health care utilization data varied considerably across different gold standard definitions. When 'RA diagnosis per rheumatologists' was used as the gold standard, the performance of all three algorithms requiring at least one DMARD prescription was acceptable, with the PPVs of 86.2% to 88.9%. Even with fulfillment of three or more of the ACR RA criteria as the gold standard, the PPVs of our algorithms were moderate to good (72.4% to 73.3%). Given the limitations of the ACR RA classification criteria for clinical practice, it may be more appropriate to use 'RA diagnosis per rheumatologists' as the gold standard.

## Conclusions

Our results indicate that, to accurately identify subjects with RA in health care utilization databases, future research should consider algorithms that link ICD-9 codes to pharmacy claim data.

## Abbreviations

ACR: American College of Rheumatology; CI: confidence interval; DMARD: disease-modifying antirheumatic drug; ICD-9: International Classification of Diseases-9th Revision; PACE: Pennsylvania Assistance Contract for the Elderly; PPV: positive predictive value; RA: rheumatoid arthritis; SD: standard deviation.

## Competing interests

DHS has received research support from Amgen (Thousand Oaks, CA, USA) and Abbott (Abbott Park, IL, USA) and support for an educational course from Bristol-Myers Squibb Company (Princeton, NJ, USA). He has non-compensation roles in two drug trials sponsored by Pfizer Inc (New York, NY, USA). The other authors declare that they have no competing interests.

## Authors' contributions

All authors participated in the study conception. AS and JMP participated in the study design and in data acquisition. JNK participated in the study design and in data analysis and interpretation. DHS participated in the study design and in data acquisition, analysis, and interpretation. SK, MEW, and HM participated in data analysis and interpretation. All authors participated in manuscript preparation and revision. All authors read and approved the final manuscript.
